# Elucidation of the mechanism of treating endometriosis with Ge Xia-Zhu Yu decoction by means of network pharmacology and molecular docking

**DOI:** 10.1097/MD.0000000000043115

**Published:** 2025-07-25

**Authors:** Shuang Li, Bo Li, Zibo Duan, Dan Liu, Xiaohua Lin

**Affiliations:** a Hebei University of Chinese Medicine, Shijiazhuang, Hebei, China; b First Affiliated Hospital, Hebei University of Chinese Medicine, Shijiazhuang, Hebei, China; c Heilongjiang University of Chinese Medicine, Ha er bin, Hei Long Jiang, China.

**Keywords:** endometriosis, Ge Xia-Zhu Yu decoction, molecular docking simulation, network pharmacology, Pharmacology of Chinese Medicine, signaling pathway

## Abstract

This study investigates the mechanism of Ge Xia-Zhu Yu decoction (GXZYT) in the treatment of endometriosis (EMS). The active components and targets of GXZYT were screened using TCMSP database and HERB database. The EMS-related genes were retrieved from Gene Cards and Disgent databases. The potential targets of GXZYT for the treatment of EMS were predicted and plotted on Venn diagrams, and construct drug-drug-target and drug-drug-target-pathway networks using Cytoscape software. Protein-protein interaction network was constructed with STRING software, and core targets were screened by CytoNCA and CytoHubb. Gene Ontology enrichment and Kyoto Encyclopedia of Genes and Genomes pathway analysis were performed on the predicted targets using the David Platform, and visualization was performed using bioinformatics. The AutoDock Vina software was used for molecular docking of key components and core targets. We obtained 165 active ingredients and 893 target genes from GXZYT and 1431 EMS-related genes in 2 disease databases. The top 5 active ingredients were quercetin, kaempferol, baicalin, tetrahydropalmatine, and luteolin, and the intersection of the top 10 core proteins were AKT1, ALB, STAT3, and TNF. Gene Ontology enrichment analysis showed that the core targets involved 904 biological processes, 117 cell components, and 218 molecular functions. Kyoto Encyclopedia of Genes and Genomes enrichment analysis showed that the core target involved 180 pathways, including the PI3K-Akt signaling pathway and other signaling pathways. The results of molecular docking showed that AKT1, ALB, STAT3, and TNF had good binding ability with quercetin, kaempferol, baicalin, tetrahydropalmatine, and luteolin. The GXZYT decoction exhibits therapeutic effects in the treatment of EMS through its anti-inflammatory, antioxidant, and anti-apoptotic effects, as well as by regulating signaling pathways such as PI3K/Akt. However, additional in vivo and clinical studies are required to validate its curative efficacy. Because the research in this article does not involve humans or animals, ethical approval was not applied for.

## 1. Introduction

Endometriosis (EMS) is a common chronic gynecological disease caused by the ectopic location of active endometrial cells outside the endometrium, it has affected about 10% of women of reproductive age worldwide. The impact of this disease is not limited to the pelvis but also affects the whole body by affecting metabolism and gene expression.^[1]^ At present, the clinical treatment of EMS can be divided into surgical treatment and drug treatment. However both have a high recurrence rate and increase the risk of infertility in patients,^[2]^ Therefore, exploring ways to treat EMS with fewer side effects is still one of the problems that the current research is looking to solve. With the in-depth study of natural botanical drugs, the use of traditional Chinese medicine (TCM) in the treatment of EMS has gradually shown its great potential.^[3]^

**Figure 1.**
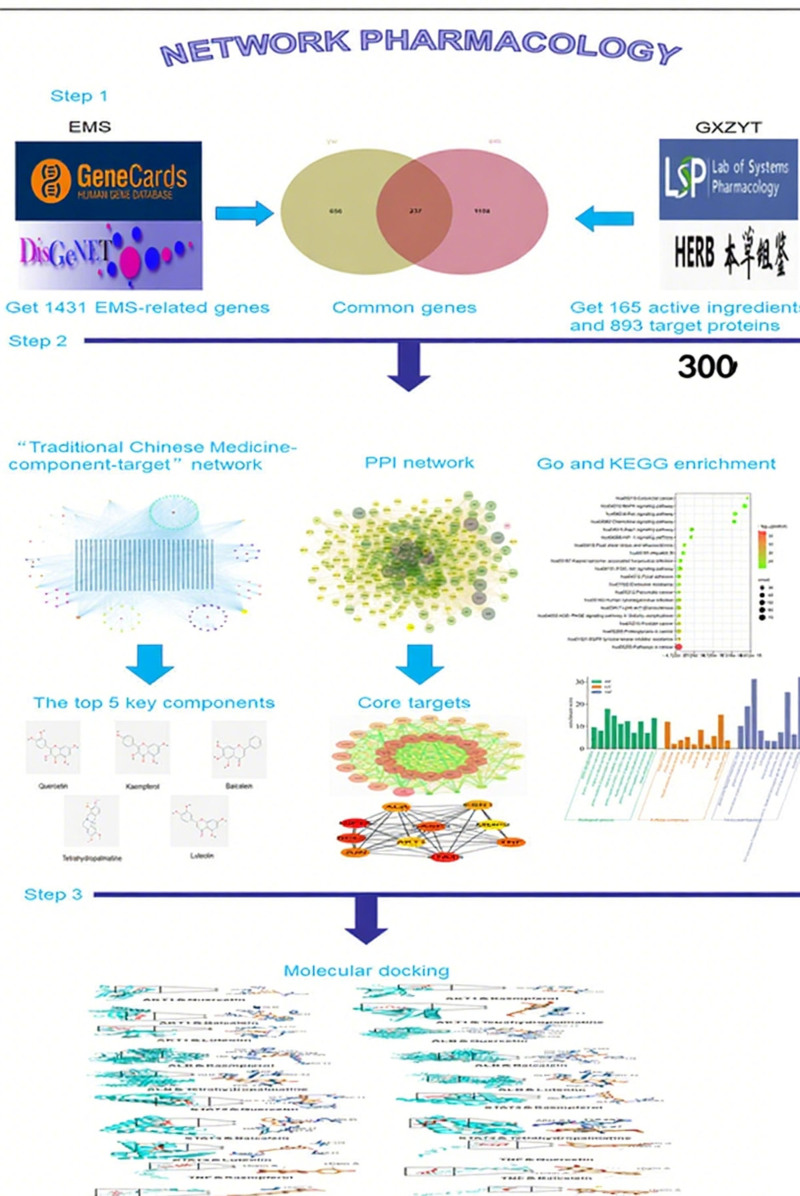
Flow chart of network pharmacology.

Ge Xia-Zhu Yu decoction (GXZYT) is a classic prescription composed of 12 kinds of drugs. With the effects of promoting blood circulation, removing blood stasis, and relieving pain, it can be used to treat metastatic or mass lesions. Metastatic disease refers to aggressive diseases such as gastric cancer. Studies have shown that GXZYT can inhibit the proliferation and metastasis of gastric cancer and promote cancer cell pyroptosis, so as to achieve the purpose of treatment.^[4]^ Mass disease refers to the substantial lesions of human organs such as liver cirrhosis, and relevant studies have shown that the effect of this prescription may play a role through multiple signaling pathways, and reduce the expression of MMP9.^[5]^ EMS has both the characteristics of tumor-like metastasis and also forms substantial lesions in ectopic tissues. Therefore, the innovation of this paper lies in exploring the mechanism of GXZYT in the treatment of EMS by using network pharmacology and molecular docking methods, aiming to provide a feasible approach for the clinical treatment of the disease. The workflow is illustrated in Figure 1

## 2. Materials and methods

### 2.1. To obtain the effective components of GXZYT

GXZYT consists of 12 TCMs, including Hong Hua, Chuan Xiong, and Tao Ren, etc. The names of 12 TCMs were recorded in TCM Systems Pharmacology Database (https://old.tcmsp-e.com/tcmsp.php),^[6]^ Screening was performed based on molecular weight ≤ 500, partition coefficient of octanol and water (AlogP) ≤ 5, hydrogen bond donor ≤ 5, hydrogen bond acceptor ≤ 10, oral bioavailability ≥ 30% and drug similarity ≥ 0.18. Then the active ingredients were screened by Swiss ADME (http://www.swissadme.ch/), and the low-active ingredients were excluded. The herb database (http:herb.ac.cn)^[7]^is used to supplement the active ingredients of Chinese medicines not included in the TCMSP database.

### 2.2. Obtain the target of GXZYT

Will obtain the effective component input Swiss target prediction^[8]^ (http://www.swisstargetprediction.ch/) in target prediction probability (likelihood > 0), and if it could not be predictable, supplement them with TCMSP. The standardization was carried out in the uniprot^[9]^ (https://www.uniprot.org) database and the species was set as “human.” The database of compounds of GXZYT and its targets was constructed.

### 2.3. Acquisition of EMS genes

Potential genes associated with EMS were determined from the human genetic database (GeneCards, https://www.genecards.org/)^[10]^ and DisGeNET database (https://www.disgenet.org/home/)^[11]^ and search words for “endometriosis,” takes 2 common parts of the database.

### 2.4. Establishment of “drug-ingredient-target” diagram

The target of GXZYT was intersected with the related genes of EMS, and the Venn diagram was drawn by bioinformatics(https://www.bioinformatics.com.cn/static/others/jvenn/example.html), so as to obtain the potential target of GXZYT in the treatment of EMS. The “traditional Chinese medicine-component-target” network was constructed by Cytoscape 3.10.1 software (Cytoscape Consortium).

### 2.5. PPI network and acquisition of core genes

In order to study the effects of target active ingredients and target proteins in GXZYT, interaction database platform STRINGv12.0 (https://cn.string-db.org/)^[12]^ was used to search target genes of drug-disease crossover, and construct protein-protein interaction (PPI) network. Set the species to “Homo sapiens” in the database search, the confidence score threshold is set to 0.4, and the other Settings are set to default. Then, cytoscape3.10.1 is used to visualize proteins and targets and analyze their interaction network. CytoNCA^[13]^ in this software is used to calculate degree, closeness, and BC values, and median value is used to screen out core proteins. Meanwhile, ten core nodes were selected by cytoHubb^[14]^ for subsequent research.

### 2.6. GO function and KEGG pathway enrichment analysis

The online platform David database (https://david.ncifcrf.gov/)^[15]^ is used for Gene Ontology (GO) annotation^[16]^ and Kyoto Encyclopedia of Genes and Genomes (KEGG) pathway enrichment analysis.^[17]^ GO categories include biological processes (BP), cellular components and molecular functions. Sort the data according to count value and *P* value, and select the top 10 of GO function and the top 20 of KEGG for visualization and analysis on online platform bioinformatics (https://www.bioinformatics.com.cn/static/others/jvenn/example.html). Cytoscape 3.10.1 was used to visualize the top 10 pathways and drugs, ingredients, and targets.

### 2.7. Molecular docking and visualization

Molecular docking is a kind of calculation based on computer structure to simulate the interrelationship between molecules and predict the interrelationship at the molecular level.^[18]^ The steps are as follows.

(1) Selection of ligands. The top 5 active ingredients were quercetin, kaempferol, baicalin, tetrahydropalmatine, and luteolin.
(2) Preparation of ligands. Download the 2D structure through the PubChem data library (https://pubchem.ncbi.nlm.nih.gov/)^[19]^ and minimize the energy through chem3D 15.1,^[20]^ then detect the root of the ligand in the AutoDock Tools 1.5.6 software (Scripps Research Institute, La Jolla) and select its rotatable bond and convert it into a 3D structure.^[21]^
(3) Selection of receptor proteins. The top ten core proteins extracted by median method were selected according to the degree value and intersected with the ten core proteins selected by CytoHubb and 4 kinds of receptor proteins were obtained.
(4) Preparation of receptor proteins. The PDB format of the receptor was downloaded from the PDB database (https://www1.rcsb.org/),^[22]^ and then the water molecules and small molecular ligands were deleted using PyMOL 3.0 software (Schrödinger LLC, New York).^[23]^ Finally, AutoDock Tools 1.5.6 software was used to hydrogenate small molecule ligands and receptors, calculate charge and debug active pockets, etc.^[21,24]^
(5) Docking of ligand and receptor proteins. The vina open source program was used for docking,^[25]^ and the binding strength and activity of the target and the active compound were evaluated based on the docking score. If the binding energy was <−5kcal/mol,^[26]^ the docking was considered feasible. Finally, PYMOL software is used for visualization.

## 3. Results

### 3.1. Screening of effective components and targets of GXZYT

The chemical components of each Chinese medicine were retrieved from the TCMSP database, and the active ingredients that met the above conditions were screened out. Since Wulingzhi was not retrieved from TCMSP, the HERB database was used to supplement it. Table 1 lists the number of active ingredients and targets in the Chinese medicines of the decoction for GXZYT screened from the TCMSP and HERB databases (note: as the effect of licorice is to reconcile various medicines, it will not be discussed.), 165 active ingredients and 893 target genes were obtained by deleting duplicates.

**Table 1:** The number of active components and action targets in GXZYT decoction.

Herbs	Active components	Active targets
Yan Hu Suo	42	3463
Wu Ling Zhi	17	565
Xiang Fu	11	957
Hong Hua	10	913
Tao Ren	10	401
Wu Yao	7	527
Chi Shao	5	108
Zhi Qiao	4	203
Dan Pi	4	234
Chuan Xiong	3	202
Dang Gui	0	0

GXZYT = Ge Xia-Zhu Yu decoction.

### 3.2. Targets related to EMS

**Figure 2.**
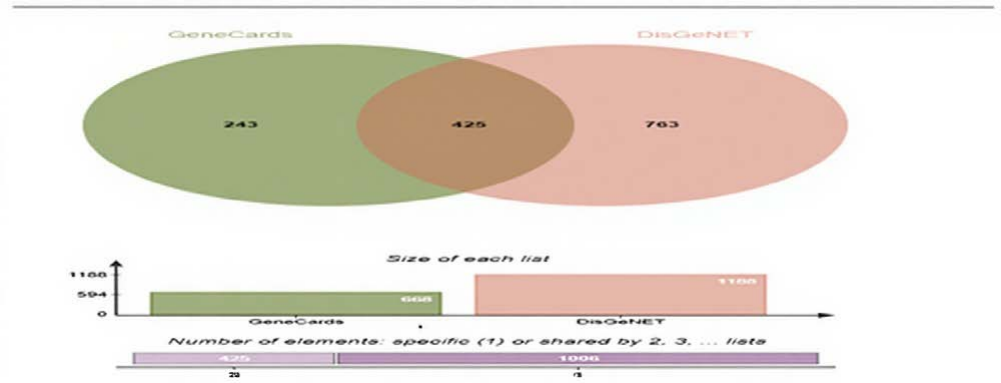
Endometriosis injury-related targets.

Based on the search method of the keyword “endometriosis,” 668 and 1188 disease targets were searched in GeneCards and DisGeNET disease databases, respectively. Then we integrated these targets and removed the duplicate values to obtain a total of 1431 EMS-related targets (see Fig. 2).

### 3.3. Prediction of potential targets for the treatment of EMS by GXZYT and establishment of the “TCM - drug target” network

**Figure 3.**
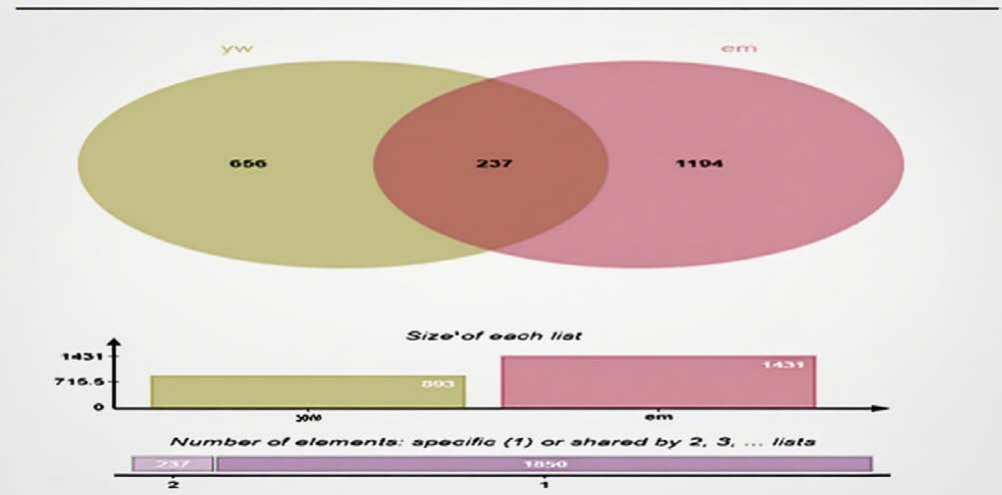
Targets prediction of GCZYT for endometriosis.

**Figure 4.**
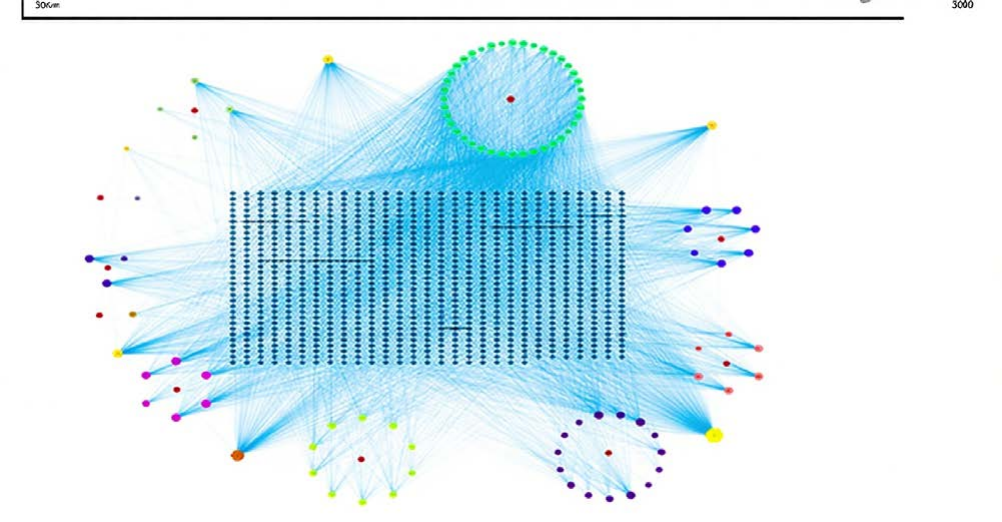
Construction of “herbal-ingredient-target” network. The larger the node degree value, the larger the shape.

**Figure 5.**
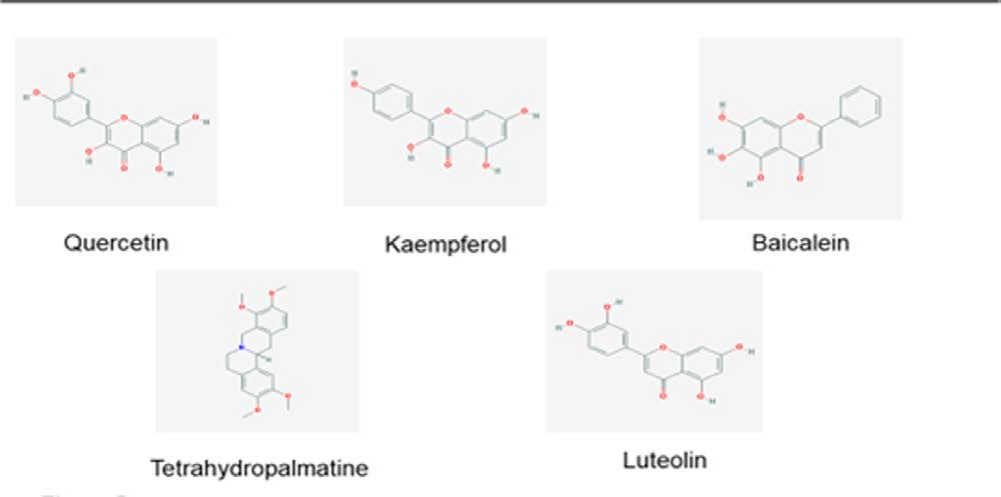
Chemical structure formulas of the top 5 active ingredients.

Two hundred thirty-seven potential targets were obtained by comparing the active ingredient targets of GXZYT with the genes related to EMS, and the Venn diagram was drawn on the bioinformatics platform (see Fig. 3, which shows the number of common targets of GXZYT and EMS), and the network of “TCM - active ingredient - predicted target” was constructed (see Fig. 4, The network relationships among TCM, active ingredients and predicted targets were shown). The first 5 active ingredients were screened, which were quercetin, kaempferol, baicalin, tetrahydropalmatine, and luteolin (see Fig. 5 and Table 2, which show the structure and related data of these 5 active ingredients).

**Table 2:** Network topological parameters of key targets.

ID	Active ingredient	Degree value
Mol 000098	Quercetin	505
Mol 000422	Kaempferol	303
Mol 002714	Baicalein	202
Mol 004071	Tetrahydropalmatine	202
Mol 000006	Luteolin	202

### 3.4. Core objectives of PPI network establishment and screening

**Figure 6.**
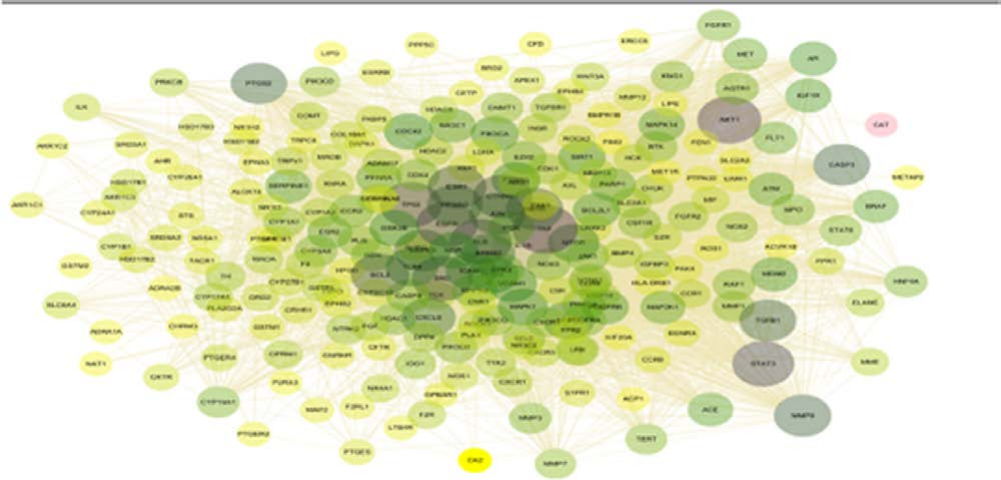
The PPI network of target proteins. The larger the node degree value, the larger the shape. PPI = protein-protein interaction.

**Figure 7.**
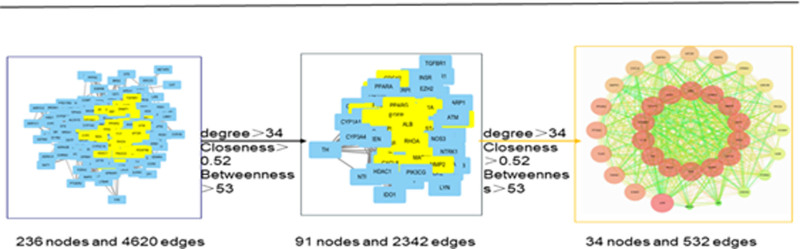
Median value method for determining core genes.

**Figure 8.**
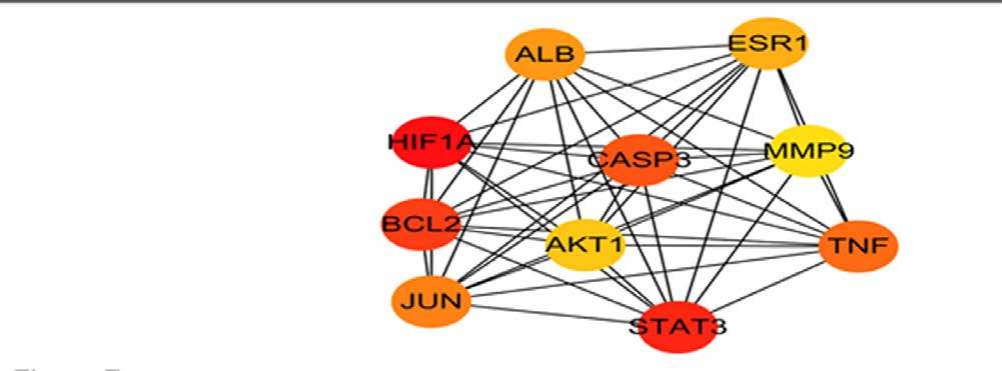
Ten core genes determined by CytoHubb.

The intersection targets of GXZYT and EMS were imported into the STRING network analysis platform to obtain the PPI protein interaction map (see Fig. 6, which shows the protein interactions between the targets). The core genes were screened using CytoNCA and CytoHubb (see Figs. 7 and 8, which show the core genes of GXZYT in the treatment of EMS).

### 3.5. GO function and KEGG pathway enrichment analysis

**Figure 9.**
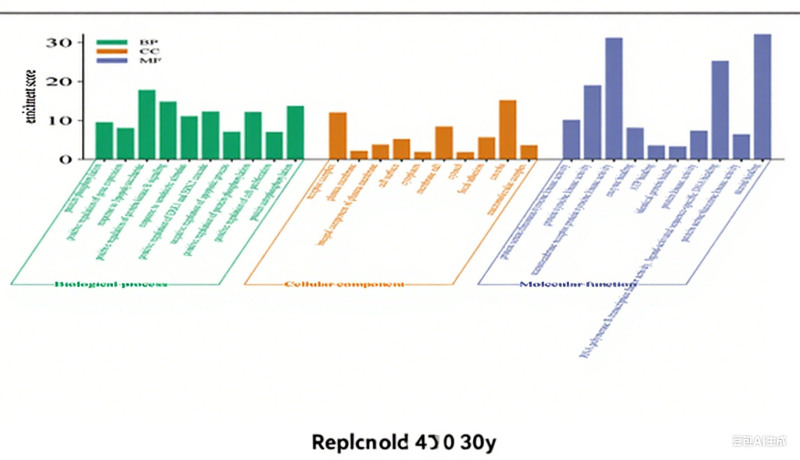
GO function enrichment analysis. GO = Gene Ontology.

**Figure 10.**
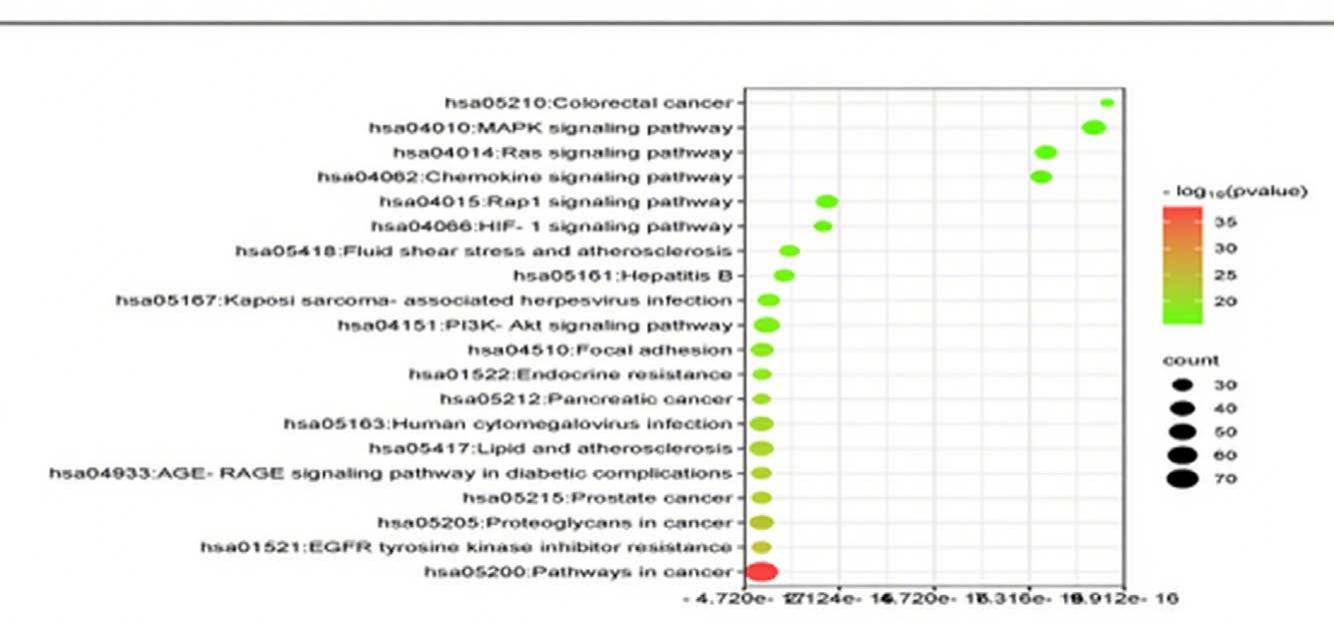
KEGG pathway enrichment analysis. KEGG = Kyoto Encyclopedia of Genes and Genomes.

**Figure 11.**
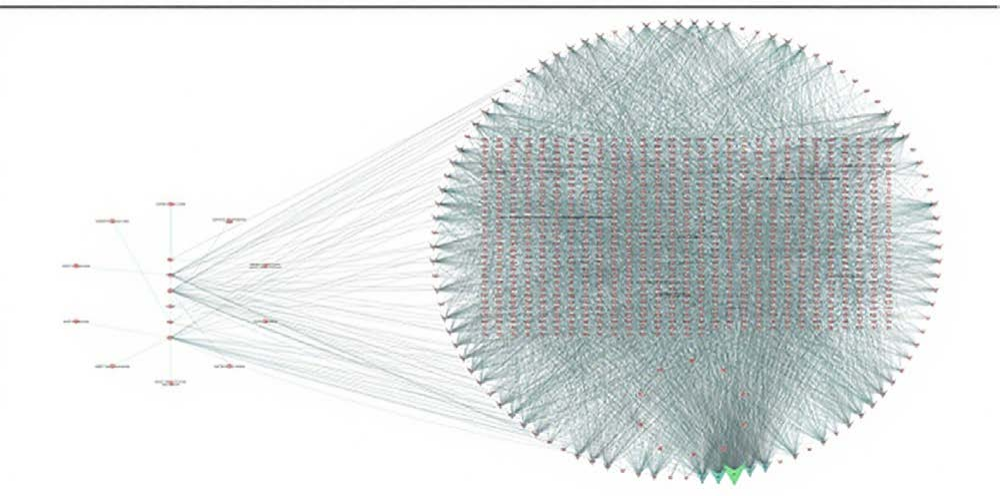
Construction of “herbal-ingredient-target-pathway” network.

The 237 predicted target factors were imported into the David bioinformation database for GO function and KEGG pathway enrichment analysis. With *P* < .05 as the screening condition, 1071 GO items and 172 KEGG pathways were obtained. GO features include 794 BP entries, 91 cellular component entries, and 186 molecular function entries. After sorting according to the *P* value, select the first ten entries to draw a bar chart. For the KEGG path, select the first 20 entries to draw a bubble chart (Figs. 9 and 10 show the visualization of the results of the screened GO and KEGG entries). The “drug-component-target-pathway” diagram is drawn with Cytoscape 3.10.1 (Fig. 11 shows the network relationship among drugs, components, targets, and pathways).

GO enrichment results showed that in BP, intersection genes were mainly related to protein phosphorylation, response to lipopolysaccharide, response to exogenous stimuli, positive regulation of ERK1 and ERK2 cascades, apoptosis, and proliferation; The results of cell component enrichment showed that the intersection genes were widely distributed in the plasma membrane, cell surface and cytoplasm; molecular functional enrichment indicated that intersection genes may affect protein tyrosine kinase activity, enzyme binding, ATP binding, DNA binding and steroid binding, etc.

KEGG pathway enrichment analysis shows that the signal pathways involved in overlapping genes include cancer-related pathways such as prostate cancer, pancreatic cancer, colorectal cancer, and cancer proteoglycans; There are also infectious disease-related pathways such as hepatitis B, Kaposi sarcoma-associated herpes virus infection, and human cytomegalovirus infection; In addition, there are metabolic disease-related pathways such as lipid and atherosclerosis and endocrine resistance; It is also involved in the reaction of PI3K-Akt signaling pathway, hypoxia-inducible factor 1 (HIF-1) signaling pathway, Rap1 signaling pathway, chemokine signaling pathway, Ras signaling pathway, MAPK signaling pathway and other signaling pathways, it shows that the decoction has multiple components, multiple targets and participates in multiple pathways.

### 3.6. Analysis of molecular docking

**Figure 12.**
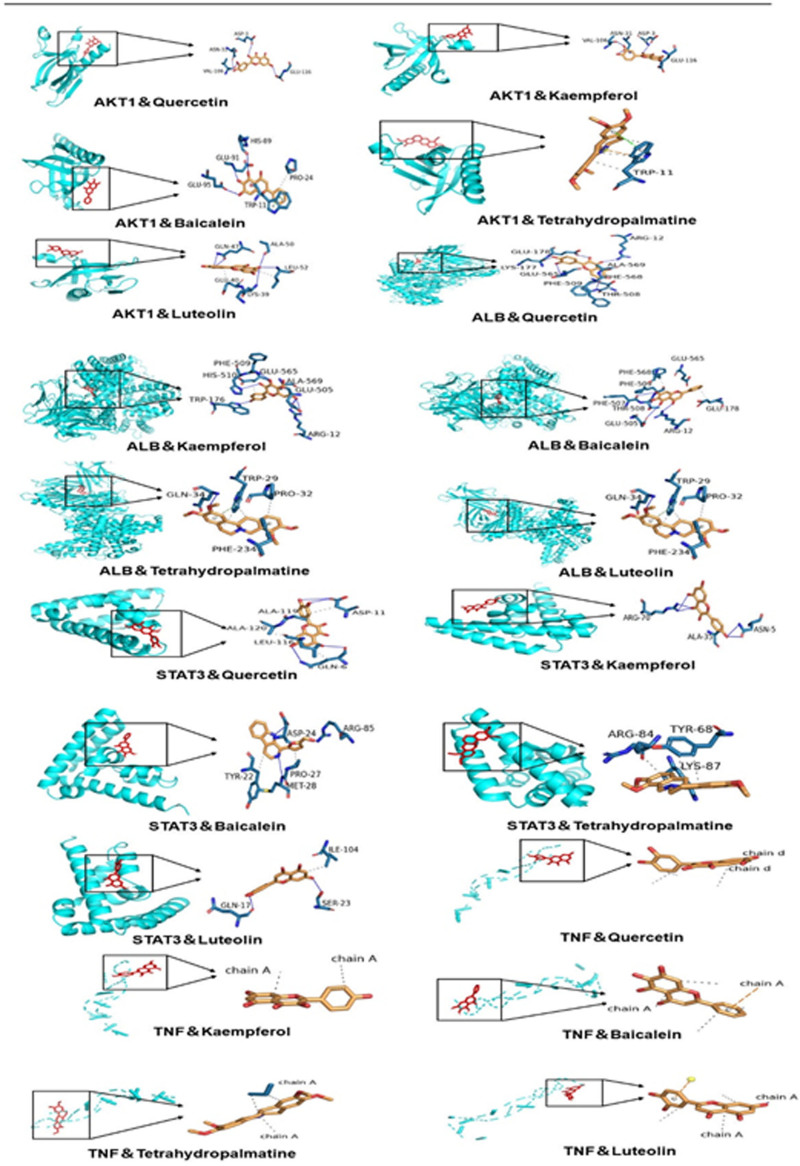
Molecular docking and binding sites visualizations.

Five main active ingredients of GXZYT, quercetin, kaempferol, baicalin, tetrahydropalmatine, and luteolin, were simulated docking with the selected core proteins AKT1, ALB, STAT3, and TNF, respectively. Binding energies < −5 kcal/mol are generally considered to be meaningful docking results (see Table 3, which shows the binding energies of the main active ingredient to the core protein). The binding energies of the 5 main active ingredients were all <−5 kcal/mol, indicating that the binding effect was good. To visualize and analyze the meaningful docking results of docking energy (see Fig. 12, showing the visualization of molecular docking and binding sites).

**Table 3:** Docking and binding ability of main active ingredients to core targets.

Binding energy (kcal/mol)					
Target	Quercetin	Kaempferol	Baicalein	Tetrahydropalmatine	Luteolin
AKT1	−6.4	−6.3	−6.8	−6.3	−6.4
ALB	−8.9	−8.8	−8.9	−8.3	−8.8
STAT3	−6.3	−6.3	−6.1	−5.6	−6.2
TNF	−6.2	−6.3	−6.3	−5.8	−6.4

## 4. Discussion

TCM has been widely used in the clinical treatment and scientific research of EMS due to its advantages of multi-effect treatment, various basic pathophysiological processes targeting diseases, and reasonable price.^[3,27,28]^ As a famous prescription through the ages, the therapeutic effect of GXZYT has been verified in clinical practice, but because of the complexity of its drug components and interactions, the specific mechanism of its treatment remained unelucidated. With the development of computational biology and pharmacology, network pharmacology, a research method with its unique advantages, has gradually emerged in the study of Chinese herbal medicine.^[29,30]^ Based on the “drug-target/molecule-protein” network pathway, network pharmacology systematically reveals the relationship between disease genes, drug active ingredients, and related proteins, from macro to micro scales,^[31,32]^ which makes the study of GXZYT more in-depth and comprehensive. GXZYT is effective in treating alcoholic fatty liver, liver fibrosis, and cirrhosis,^[5,33,34]^ The clinical application of GXZYT in the adjuvant treatment of advanced pancreatic cancer patients has shown better efficacy and survival rates, which may be because Chinese herbal therapy has less toxic side effects than chemotherapy, and relevant experiments have shown that the addition of Chinese herbal medicine can inhibit the proliferation and induce the apoptosis.^[35]^ GXZYT has therapeutic effects on metastatic and mass diseases,^[4,5]^ but its mechanism of action in EMS remains unclear.

Network pharmacological analysis showed that the main active ingredients of GXZYT were (+) -catechin, quercetin, kaempferol, baicalin, tetrahydropalmatine, and luteolin. The results of GO enrichment analysis showed that the target genes regulated by the decoction might be distributed in the plasma membrane, cell surface, and cytoplasm, and participate in the regulation of cell proliferation and apoptosis; KEGG pathway analysis showed that GXZYT decoction was involved in the regulation of PI3K-Akt signaling pathway, HIF-1 signaling pathway, Ras (Rap1) signaling pathway and chemokines signaling pathway, MAPK signaling pathway.

The PI3K/AKT signaling pathway is an important regulatory pathway involved in cell growth, metabolism, apoptosis, and other activities.^[36]^ Relevant studies have shown that luteolin can inhibit cell viability, migration, angiogenesis, and invasion by activating the PI3K/AKT signaling pathway, which may play a therapeutic role in ectopic endometrium.^[37,38]^ HIF-1 is one of the most characteristic oxygen-regulated transcriptional activators of the HIF family, which can be divided into 2 subtypes: α and β. Both mTOR pathway and extracellular signal-regulated kinase (ERK)-mediated phosphorylation enhance the transcription activity of HIF-1, and NF-κB pathway contributes to the maintenance of HIF1α mRNA levels.^[39]^ Rap1 is a member of the RAS-like small GTP-binding protein family and also the most conserved telomere interaction protein. Relevant studies have shown that activation of Rap1GAP can inhibit the proliferation, metastasis, and invasion of tumor cells, characteristics also shared by EMS.^[40,41]^ Chemokines are a general term for a class of small cytokines or signaling proteins. Reduced toxicity of NK cells will promote the onset of EMS, and CXCL1 can promote the expression of NK cells by activating PKD2/mTOR signaling pathway, thus achieving the purpose of treating EMS.^[42,43]^ MAPK signaling kinases can be divided into 3 types: ERK, p38, and c-jun terminal kinase. Although ERK is the most widely studied, the combination of upstream molecules of p38 and c-jun terminal kinase and the reduction of its related side effects by downstream targeting provide directions for the treatment of EMS.^[44,45]^ Some related studies have shown that baicalin can regulate the expression of IL-6, IL-1 β, tumor necrosis factor-α (TNF-α), MIP-2, and MIP-1 α through PI3K/Akt/NRF2 and other pathways, thus regulating the inflammatory response induced by oxidative stress.^[46]^ The flavonoid kaempferol and quercetin are natural products that directly bind to nuclear receptor 4A1 (NR4A1), and they can inhibit the proliferation of human endometrial epithelial cells and Ishikawa cells by inhibiting epidermal growth factor receptors and other pathways.^[47]^ However, the mechanism of these drugs needs to be further studied.

The results of molecular docking showed that the main active ingredients, including quercetin, kaempferol, baicalin, tetrahydropalmatine, and luteolin had good docking abilities with the target proteins AKT1, ALB, STAT3, and TNF. AKT1 is a member of the AKT family, which plays an important role in cell cycle control, apoptosis evasion metabolism, etc. During the pathogenesis of EMS, AKT1 expression increases. Drugs that reduce AKT1 expression can not only inhibit the proliferation of ectopic endometrial cells but also improve fertility.^[48]^ ALB is a non-glycoprotein composed of a single polypeptide chain, which has high structural stability and is known as the most abundant protein in extracellular fluid. ALB can interact with >20 pathways, and in the connection of the apical pathway, it has been found that the high expression of uPAR may have endowed essential protein functions in the molecular mechanism of invasion and metastasis. Therefore, ALB may play a role in inhibiting cell invasion and metastasis through uPAR.^[49]^ STAT3 can be divided into 3 isomers, among which the SH2 domain can specifically recognize phosphorylated tyrosine residues and thus be activated by phosphorylation. The phosphorylated STAT3 can enter the nucleus and bind to the promoter of the target gene to activate transcription. Relevant in vivo and in vitro experiments have shown that reducing the signaling and phosphorylation of STAT3 can alleviate the adhesion and volume of EMS.^[50]^ TNF-α is a pleiotropic inflammatory cytokine in the TNF ligand superfamily (TNFSF), produced by a variety of immune cells,^[51]^ In EMS cells, TNF-α can be upregulated, which increases the phosphorylation of PI3K, AKT, ERK, and NF-κB signaling pathways, and then the dysregulation of MiRNA expression promotes the pathological development of EMS.^[52]^ Inhibition of TNF-α upregulation may inhibit the course of EMS. In conclusion, core target proteins AKT1, ALB, STAT3, and TNF all play important roles in the occurrence and development of EMS. However, how quercetin, kaempferol, baicalin, tetrahydropalmatine, and luteolin, which have good docking effects with the core target proteins, regulate their expression remains to be further verified by experiments In conclusion, the network pharmacology offers an approach to the specific pharmacological mechanism of the treatment of EMS by GXZYT.

## 5. Conclusion

Therefore, the treatment of EMS by GXZYT is a synergistic process with multiple active ingredients, multiple targets, and multiple pathways. The results showed that the active components of the compound, such as quercetin, kaempferol, baicalin, tetrahydropalmatine, and luteolin, may act on target proteins such as AKT1, ALB, STAT3, and TNF, and regulate I to regulate PI3K-Akt signaling pathway, HIF-1 signaling pathway, Ras (Rap1) signaling pathway. Chemokine signaling pathway and MAPK signaling pathway play a role in improving EMS by inhibiting cell proliferation and invasion, inhibiting oxidative stress, and inhibiting inflammatory response. However, the exact mechanism needs to be verified by animal or clinical studies, which we will continue to explore in follow-up studies.

## Acknowledgments

Thanks to all the authors who contributed to the study.

## Author contributions

**Conceptualization:** Shuang Li, Bo Li, Zibo Duan, Dan Liu, Xiaohua Lin.

**Data curation:** Shuang Li.

**Formal analysis:** Zibo Duan.

**Methodology:** Bo Li.

**Software:** Bo Li.

**Validation:** Dan Liu, Xiaohua Lin.

**Visualization:** Shuang Li.

**Writing – original draft:** Shuang Li.

**Writing – review & editing:** Zibo Duan, Dan Liu, Xiaohua Lin.
